# Crystal structures of the co-crystalline adduct 5-(4-bromo­phen­yl)-1,3,4-thia­diazol-2-amine–4-nitro­benzoic acid (1/1) and the salt 2-amino-5-(4-bromo­phen­yl)-1,3,4-thia­diazol-3-ium 2-carb­oxy-4,6-di­nitro­phenolate

**DOI:** 10.1107/S1600536814021138

**Published:** 2014-10-04

**Authors:** Graham Smith, Daniel E. Lynch

**Affiliations:** a Science and Engineering Faculty, Queensland University of Technology, GPO Box 2434, Brisbane, Queensland 4001, Australia; b Exilica Limited, The Technocentre, Puma Way, Coventry, CV1 2TT, England

**Keywords:** 5-substituted-2-amino-1,3,4-thia­diazo­les, 4-nitro­benzoic acid, 3,5-di­nitro­salicylic acid, proton transfer, mol­ecular adducts, co-crystals, hydrogen bonding, π–π inter­actions, crystal structure

## Abstract

The structures of the 1:1 co-crystalline adduct C_8_H_6_BrN_3_S·C_7_H_5_NO_4_, (I), and the salt C_8_H_7_BrN_3_S^+^·C_7_H_3_N_2_O_7_^−^, (II), obtained from the inter­action of 5-(4-bromo­phen­yl)-1,3,4-thia­diazol-2-amine with 4-nitro­benzoic acid and 3,5-di­nitro­salicylic acid, respectively, have been determined. The primary inter-species association in both (I) and (II) is through duplex *R* ^2^_2_(8) (N—H⋯O/O—H⋯O) or (N—H⋯O/N—H⋯O) hydrogen bonds, respectively, giving heterodimers. In (II), these are close to planar [the dihedral angles between the thia­diazole ring and the two phenyl rings are 2.1 (3) (intra) and 9.8 (2)° (inter)], while in (I) these angles are 22.11 (15) and 26.08 (18)°, respectively. In the crystal of (I), the heterodimers are extended into a chain along *b* through an amine N—H⋯N_thia­diazole_ hydrogen bond but in (II), a centrosymmetric cyclic hetero­tetra­mer structure is generated through N—H⋯O hydrogen bonds to phenol and nitro O-atom acceptors and features, together with the primary *R* ^2^_2_(8) inter­action, conjoined *R* ^4^_6_(12), *R* ^2^_1_(6) and *S*(6) ring motifs. Also present in (I) are π–π inter­actions between thia­diazole rings [minimum ring-centroid separation = 3.4624 (16) Å], as well as short Br⋯O_nitro_ inter­actions in both (I) and (II) [3.296 (3) and 3.104 (3) Å, respectively].

## Abstract 2

The hydrogen-bonded structures of both a (1:1) mol­ecular adduct and a salt of 5-(4-bromo­phen­yl)-1,3,4-thia­diazol-2-amine with 4-nitro­benzoic acid and 3,5-di­nitro­salicylic acid, respectively, have been determined.

## Chemical context   

1,3,4-Thia­diazole (TZ) and its derivatives, particularly the 2-amino-substituted analogues (ATZ), which are commonly phenyl-substituted at the 5-site of the thia­diazole ring, exhibit a broad range of biological activities (Jain *et al.*, 2013 ▶). In the solid state, these 2-amino-1,3,4-thia­diazo­les usually inter­act through duplex N—H⋯N hydrogen bonds, giving a centrosymmetric cyclic 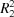
(8) hydrogen-bonding homodimer motif, which may be discrete *e.g.* the 5-(3-fluoro­phen­yl)-ATZ deriv­ative (Wang *et al.*, 2009 ▶) or more often is extended into a one-dimensional chain structure through the second 2-amino H-atom by an N—H⋯N4_thia­diazole_ hydrogen bond, *e.g.* in the 5-(4-bromo­phen­yl)-ATZ derivative (Lynch, 2009*a* ▶) and the 5-(4-bromo-2-nitro­phen­yl)-ATZ derivative (Zhang *et al.*, 2011 ▶).

With an inter­est in the formation of co-crystalline adducts as opposed to proton-transfer salt formation between Lewis bases and aromatic carb­oxy­lic acids, we have looked at some of these 5-phenyl-substituted ATZ analogues and have reported examples of both structure types: one-dimensional chain structures in the 1:1 adduct of 5-(4-meth­oxy­phen­yl)-2-amino-1,3,4-thia­diazol-2-amine with 4-nitro­benzoic acid (Lynch, 2009*b* ▶) and 5-(4-bromo­phen­yl)-2-amino-1,3,4-thia­diazol-2-amine (BATZ) with 2-(naphthalen-2-yl­oxy)acetic acid (Smith & Lynch, 2013 ▶), as well as the salt of BATZ with 3,5-di­nitro­benzoic acid (Smith & Lynch, 2013 ▶). In this salt structure, the carboxyl­ate group gives the previously mentioned primary cyclic 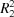
(8) association through carboxyl O⋯H—N and amine N—H⋯O hydrogen bonds but instead of forming the chain structure, a centrosymmetric hetero­tetra­mer is formed through a cyclic 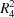
(8) hydrogen-bonding motif.
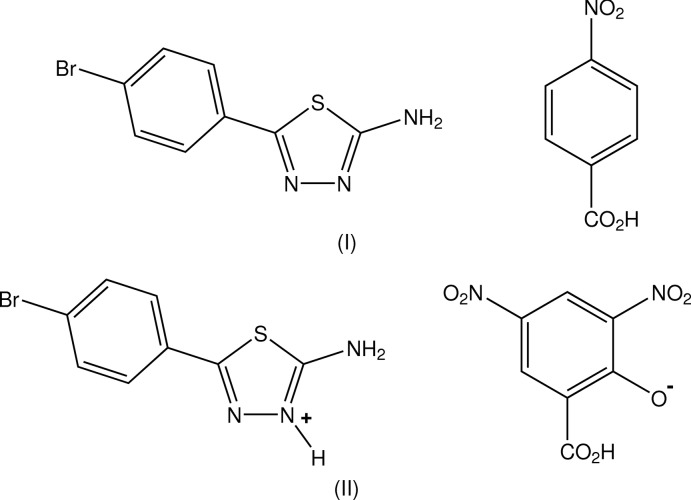


Herein we report the structures of the 1:1 co-crystalline adduct, C_8_H_6_BrN_3_S·C_7_H_5_NO_4_, (I), and the salt C_8_H_7_BrN_3_S^+^·C_7_H_3_N_2_O_7_^−^, (II), obtained from the inter­action of BATZ with 4-nitro­benzoic acid (PNBA) and 3,5-di­nitro­salicylic acid (DNSA), respectively. The strong acid DNSA (p*K*_a_ = 2.18) has been employed extensively for the formation of crystalline salts with Lewis bases, forming mainly phenolates (Smith *et al.*, 2007 ▶), whereas the weaker acid PNBA (p*K*_a_ = 3.44) provides examples of both salts (Byriel *et al.*, 1992 ▶) and co-crystalline adducts (Aakeröy *et al.*, 2004 ▶).

## Structural commentary   

**Figure 1:**
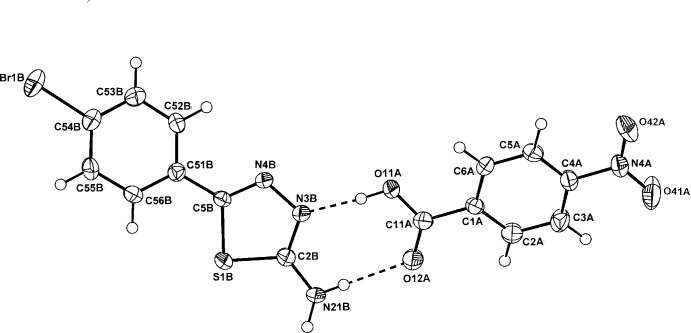
Mol­ecular conformation and atom-numbering scheme for adduct (I), with inter-species hydrogen bonds shown as dashed lines. Non-H atoms are shown as 50% probability displacement ellipsoids.

In the structure of the (1:1) PNBA adduct with BATZ, (I), the primary inter-species 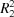
(8) hydrogen-bonded heterodimer is formed (Fig. 1 ▶), in which the 4-bromo­phenyl ring substituent is rotated slightly out of the thia­diazole plane [dihedral angles between the thia­diazole ring and the two benzene rings are 22.11 (15) (intra) and 26.08 (18)° (inter)]. The carb­oxy­lic acid and nitro substituent groups on the PNBA mol­ecule are rotated slightly out of the benzene plane [torsion angles: C2*A*—C1*A*—C11*A*—O11*A* = −170.2 (3) and C3*A*— C4*A*—N4*A*—O42*A* = 172.03 (3)°]. This ‘planar’ conformation is found in the parent acid (Bolte, 2009 ▶) and in its adducts, *e.g.* with 3-(*N,N*-di­methyl­amino)­benzoic acid (Aakeröy *et al.*, 2004 ▶).

**Figure 2:**
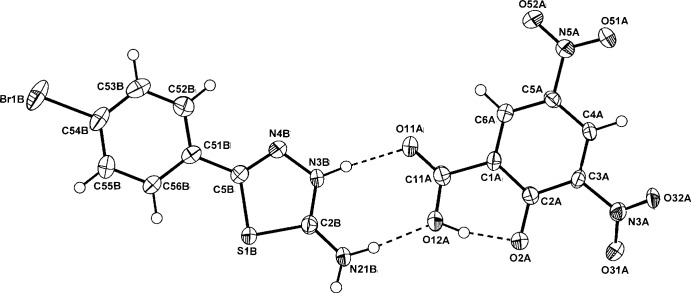
Mol­ecular conformation and atom-numbering scheme for salt (II), with inter-species hydrogen bonds shown as dashed lines. Non-H atoms are shown as 50% probability displacement ellipsoids.

In the DNSA salt (II) (Fig. 2 ▶), the primary association is also the expected cyclic 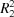
(8) heterodimer, which is essentially planar [comparative dihedral angles 9.8 (2) (intra) and 2.1 (2)° (inter)]. The DNSA anionic moiety is a phenolate with the *anti*-related carb­oxy­lic acid H atom forming the common intra­molecular *S*(6) hydrogen bond which is found in *ca.* 70% of DNSA salt structures (Smith *et al.*, 2007 ▶). The nitro group at C3*A* in this anion is rotated significantly out of the benzene plane [torsion angle: C2*A*—C3*A*—N3*A*—O32*A* = −147.8 (4)°] whereas the second nitro group and the carboxyl­ate group lie essentially in the plane [torsion angles: C6*A*—C5*A*— N5*A*—O51*A* = 179.5 (4) and C2*A*—C1*A*— C11*A*—O11*A* = −178.0 (4)°].

## Supra­molecular features   

**Figure 3:**
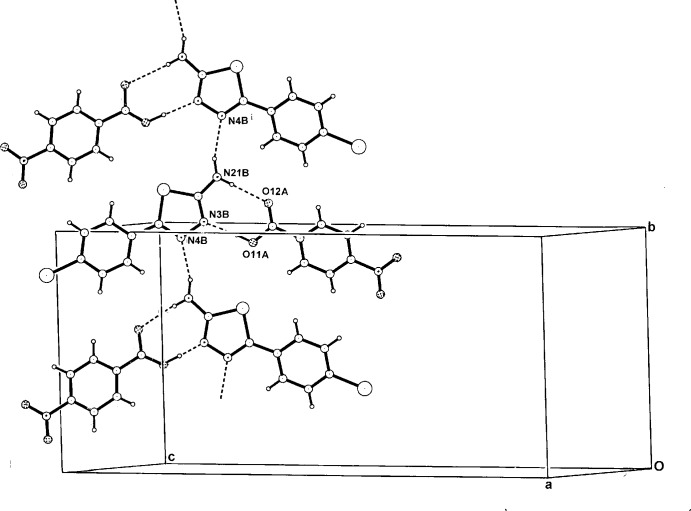
A perspective view of the one-dimensional hydrogen-bonded extension in the structure of (I). Hydrogen bonds are shown as dashed lines.

In (I), the heterodimers are linked through amine N21*B*—H21*B*⋯N4*B* ^i^ hydrogen bonds (Table 1 ▶) forming chains which extend along *b* (Fig. 3 ▶). This is similar to the structure of the BATZ adduct with 2-naphthoxyacetic acid (Smith & Lynch, 2013 ▶) and the 5-(4-meth­oxy­phen­yl)thia­diazin-2-amine adduct with 4-NBA (Lynch, 2009*b* ▶). A weak aromatic C55*B*—H55*B*⋯O41*A* ^ii^ hydrogen-bonding association links the chains across *c* [for symmetry codes, see Table 1 ▶] and together with π–π inter­actions between thia­diazole rings [minimum ring-centroid separation = 3.4624 (16) Å], give a two-dimensional supra­molecular structure.

**Figure 4:**
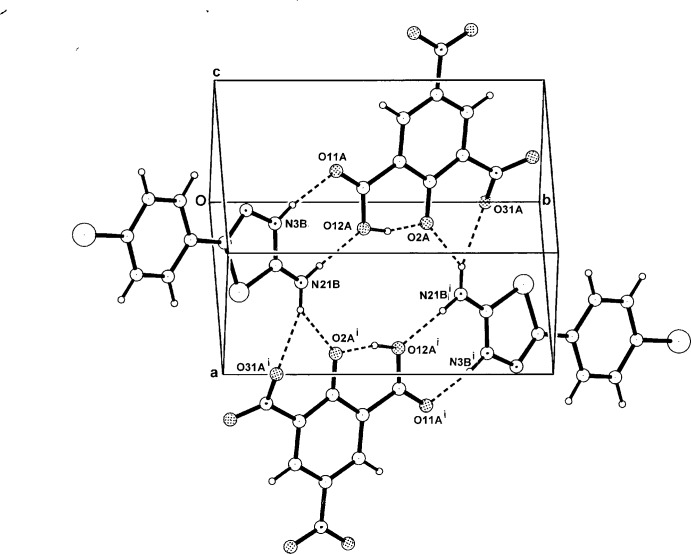
A perspective view of the centrosymmetric hydrogen-bonded hetero­tetra­mer units in the unit cell of (II), showing conjoined cyclic 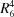
(12), 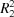
(8), 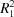
(6) and *S*(6) hydrogen-bonded structural motifs.

With (II), a secondary symmetric three-centre hydrogen-bonding inter­action between the second amine-H atom and both the phenolate-O atom (O2*B*) and the adjacent nitro-O atom (O31*A*) (Table 2 ▶) gives an enlarged centrosymmetric cyclic 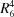
(12) association. This generates a hetero­tetra­mer, which comprises a total of seven conjoined cyclic motifs, the central 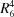
(12) plus two each of 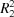
(8), 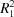
(6) and *S*(6) motifs (Fig. 4 ▶). The hetero­tetra­mers are weakly linked peripherally through both a centrosymmetric cyclic C—H⋯O_nitro_ [C4*A*—H4*A*⋯O32*A* ^ii^] hydrogen-bond pair [graph set 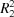
(10)] and a linear C56*B*—H56*B*⋯O51*A* ^iii^ hydrogen bond, giving a two-dimensional supra­molecular structure (for symmetry codes, see Table 2 ▶). Within the cyclic association there is a short O32*A*⋯O32*A* ^ii^ non-bonding contact [2.835 (4) Å]. However, unlike in the structure of (I), no π–π ring inter­actions are found in (II) [minimum ring-centroid separation = 4.078 (3) Å].

In both (I) and (II), short Br⋯O_nitro_ contacts are found: for (I) Br1*B*⋯O42*A* ^iii^ = 3.314 (4) Å, and for (II), Br1*B*⋯ O52*A* ^iv^ = 3.104 (3) Å [symmetry codes: (iii) *x* + 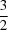, −*y* + 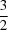, *z* + 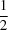; (iv) −*x*, −*y*, −*z* + 1].

## Synthesis and crystallization   

The title compounds were prepared by the reaction of 1 mmol (260 mg) of 5-(4-bromo­phen­yl)-1,3,4-thia­diazol-2-amine with 1 mmol of either 4-nitro­benzoic acid (167 mg) [for (I)] or 3,5-di­nitro­salicylic acid (228 mg) [for (II)] in 20 mL of 50% ethanol–water, with 10 min refluxing. Partial evaporation of the solvent gave colourless needles of (I) or yellow plates of (II) from which specimens were cleaved for the X-ray analyses.

## Refinement details   

Crystal data, data collection and structure refinement details are summarized in Table 3 ▶. Hydrogen atoms potentially involved in hydrogen-bonding inter­actions were located by difference methods but were subsequently included in the refinements with positional parameters fixed and their isotropic displacement parameters riding, with *U*_iso_(H) = 1.2*U*_eq_(N) or 1.5*U*_eq_(O). Other H atoms were included at calculated positions [C—H = 0.95 Å] and also treated as riding, with *U*_iso_(H) = 1.2*U*_eq_(C).

## Supplementary Material

Crystal structure: contains datablock(s) global, I, II. DOI: 10.1107/S1600536814021138/lh5731sup1.cif

Structure factors: contains datablock(s) I. DOI: 10.1107/S1600536814021138/lh5731Isup2.hkl

Structure factors: contains datablock(s) II. DOI: 10.1107/S1600536814021138/lh5731IIsup3.hkl

Supporting information file. DOI: 10.1107/S1600536814021138/lh5731Isup4.cml

Supporting information file. DOI: 10.1107/S1600536814021138/lh5731IIsup5.cml

CCDC references: 1025540, 1025541

Additional supporting information:  crystallographic information; 3D view; checkCIF report

## Figures and Tables

**Table 1:** Hydrogen-bond geometry (, ) for (I)

*D*H*A*	*D*H	H*A*	*D* *A*	*D*H*A*
O11*A*H11*A*N3*B*	0.90	1.75	2.648(3)	175
N21*B*H21*B*O12*A*	0.82	2.04	2.859(4)	172
N21*B*H22*B*N4*B* ^i^	0.92	2.16	3.052(3)	162
C55*B*H55*B*O41*A* ^ii^	0.95	2.47	3.302(4)	146

Symmetry codes: (i) 
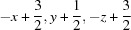
; (ii) 
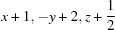
.

**Table 2:** Hydrogen-bond geometry (, ) for (II)

*D*H*A*	*D*H	H*A*	*D* *A*	*D*H*A*
O12*A*H12*A*O2*A*	0.87	1.57	2.418(4)	164
N3*B*H3*B*O11*A*	0.88	1.87	2.744(4)	172
N21*B*H21*B*O12*A*	0.88	1.89	2.747(4)	166
N21*B*H22*B*O2*A* ^i^	0.88	2.22	2.897(4)	134
N21*B*H22*B*O31*A* ^i^	0.88	2.19	2.986(5)	150
C4*A*H4*A*O32*A* ^ii^	0.95	2.44	3.284(5)	148
C56*B*H56*B*O51*A* ^iii^	0.95	2.44	3.364(5)	164

Symmetry codes: (i) 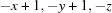; (ii) 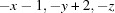; (iii) 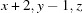.

**Table 3:** Experimental details

	(I)	(II)
Crystal data		
Chemical formula	C_8_H_6_BrN_3_SC_7_H_5_NO_4_	C_8_H_7_BrN_3_S^+^C_7_H_3_N_2_O_7_
*M*_r_	423.25	484.25
Crystal system, space group	Monoclinic, *C*2/*c*	Triclinic, *P* 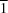
Temperature (K)	200	200
*a*, *b*, *c* ()	8.5205(6), 12.0394(7), 31.4321(18)	5.8017(3), 10.1903(5), 15.1592(9)
, , ()	90, 92.982(6), 90	88.884(4), 82.438(5), 85.470(4)
*V* (^3^)	3220.0(3)	885.62(8)
*Z*	8	2
Radiation type	Mo *K*	Mo *K*
(mm^1^)	2.71	2.49
Crystal size (mm)	0.30 0.10 0.05	0.25 0.20 0.18

Data collection		
Diffractometer	Oxford Diffraction Gemini-S CCD detector	Oxford Diffraction Gemini-S CCD detector
Absorption correction	Multi-scan (*CrysAlis PRO*; Agilent, 2013 ▶)	Multi-scan (*CrysAlis PRO*; Agilent, 2013 ▶)
*T*_min_, *T*_max_	0.936, 0.980	0.903, 0.980
No. of measured, independent and observed [*I* > 2(*I*)] reflections	6234, 3164, 2446	5742, 3458, 2479
*R*_int_	0.029	0.045
(sin /)_max_ (^1^)	0.617	0.617

Refinement		
*R*[*F* ^2^ > 2(*F* ^2^)], *wR*(*F* ^2^), *S*	0.044, 0.093, 1.05	0.058, 0.134, 1.08
No. of reflections	3164	3458
No. of parameters	226	263
H-atom treatment	H-atom parameters constrained	H-atom parameters constrained
_max_, _min_ (e ^3^)	0.37, 0.30	0.78, 0.82

Computer programs: *CrysAlis PRO* (Agilent, 2013 ▶), *SIR92* (Altomare *et al.*, 1993 ▶), *SHELXL97* (Sheldrick, 2008 ▶) within *WinGX* (Farrugia, 2012 ▶) and *PLATON* (Spek, 2009 ▶).

## supplementary crystallographic information

### Crystal data

**Table:**

C_8_H_7_BrN_3_S^+^·C_7_H_3_N_2_O_7_^−^	*Z* = 2
*M**_r_* = 484.25	*F*(000) = 484
Triclinic, *P*1	*D*_x_ = 1.816 Mg m^−^^3^
Hall symbol: -P 1	Mo *K*α radiation, λ = 0.71073 Å
*a* = 5.8017 (3) Å	Cell parameters from 1277 reflections
*b* = 10.1903 (5) Å	θ = 3.6–24.8°
*c* = 15.1592 (9) Å	µ = 2.49 mm^−^^1^
α = 88.884 (4)°	*T* = 200 K
β = 82.438 (5)°	Block, yellow
γ = 85.470 (4)°	0.25 × 0.20 × 0.18 mm
*V* = 885.62 (8) Å^3^	

### Data collection

**Table:**

Oxford Diffraction Gemini-S CCD detector diffractometer	3458 independent reflections
Radiation source: Enhance (Mo) X-ray source	2479 reflections with *I* > 2σ(*I*)
Graphite monochromator	*R*_int_ = 0.045
Detector resolution: 16.077 pixels mm^-1^	θ_max_ = 26.0°, θ_min_ = 3.4°
ω scans	*h* = −7→7
Absorption correction: multi-scan (*CrysAlis PRO*; Agilent, 2013)	*k* = −11→12
*T*_min_ = 0.903, *T*_max_ = 0.980	*l* = −18→9
5742 measured reflections	

### Refinement

**Table:**

Refinement on *F*^2^	Secondary atom site location: difference Fourier map
Least-squares matrix: full	Hydrogen site location: inferred from neighbouring sites
*R*[*F*^2^ > 2σ(*F*^2^)] = 0.058	H-atom parameters constrained
*wR*(*F*^2^) = 0.134	*w* = 1/[σ^2^(*F*_o_^2^) + (0.0545*P*)^2^] where *P* = (*F*_o_^2^ + 2*F*_c_^2^)/3
*S* = 1.08	(Δ/σ)_max_ < 0.001
3458 reflections	Δρ_max_ = 0.78 e Å^−^^3^
263 parameters	Δρ_min_ = −0.82 e Å^−^^3^
0 restraints	Extinction correction: *SHELXL97*, FC^*^=KFC[1+0.001XFC^2^Λ^3^/SIN(2Θ)]^-1/4^
Primary atom site location: structure-invariant direct methods	Extinction coefficient: 0.042 (3)

### Special details

**Table:**

Geometry. Bond distances, angles *etc*. have been calculated using the rounded fractional coordinates. All su's are estimated from the variances of the (full) variance-covariance matrix. The cell e.s.d.'s are taken into account in the estimation of distances, angles and torsion angles
Refinement. Refinement of *F*^2^ against ALL reflections. The weighted *R*-factor *wR* and goodness of fit *S* are based on *F*^2^, conventional *R*-factors *R* are based on *F*, with *F* set to zero for negative *F*^2^. The threshold expression of *F*^2^ > σ(*F*^2^) is used only for calculating *R*-factors(gt) *etc*. and is not relevant to the choice of reflections for refinement. *R*-factors based on *F*^2^ are statistically about twice as large as those based on *F*, and *R*- factors based on ALL data will be even larger.

### Fractional atomic coordinates and isotropic or equivalent isotropic displacement parameters (Å^2^)

**Table:**

	*x*	*y*	*z*	*U*_iso_*/*U*_eq_	
O2A	0.1351 (5)	0.6524 (3)	0.0207 (2)	0.0330 (9)	
O11A	−0.0271 (5)	0.3833 (3)	0.2158 (2)	0.0373 (10)	
O12A	0.2276 (5)	0.4583 (3)	0.1075 (2)	0.0374 (10)	
O31A	−0.0556 (5)	0.8407 (3)	−0.0818 (2)	0.0360 (10)	
O32A	−0.2559 (5)	0.9897 (3)	0.0027 (2)	0.0367 (10)	
O51A	−0.8459 (5)	0.8277 (3)	0.2177 (2)	0.0434 (11)	
O52A	−0.7757 (5)	0.6493 (3)	0.2912 (2)	0.0447 (11)	
N3A	−0.1754 (6)	0.8761 (4)	−0.0119 (2)	0.0298 (11)	
N5A	−0.7202 (6)	0.7301 (4)	0.2328 (2)	0.0314 (12)	
C1A	−0.1349 (7)	0.5803 (4)	0.1393 (3)	0.0245 (12)	
C2A	−0.0684 (7)	0.6698 (4)	0.0697 (3)	0.0255 (12)	
C3A	−0.2287 (7)	0.7776 (4)	0.0571 (3)	0.0256 (12)	
C4A	−0.4397 (7)	0.7983 (4)	0.1105 (3)	0.0248 (12)	
C5A	−0.4948 (7)	0.7078 (4)	0.1777 (3)	0.0251 (12)	
C6A	−0.3473 (7)	0.5999 (4)	0.1929 (3)	0.0274 (12)	
C11A	0.0285 (7)	0.4637 (4)	0.1578 (3)	0.0294 (14)	
Br1B	0.60639 (11)	−0.41426 (5)	0.59418 (3)	0.0574 (2)	
S1B	0.68942 (18)	0.05777 (11)	0.23777 (7)	0.0314 (3)	
N3B	0.2976 (6)	0.1838 (3)	0.2484 (2)	0.0288 (11)	
N4B	0.2693 (6)	0.0962 (3)	0.3171 (2)	0.0303 (11)	
N21B	0.5551 (6)	0.2544 (3)	0.1290 (2)	0.0346 (11)	
C2B	0.5050 (7)	0.1784 (4)	0.1978 (3)	0.0267 (12)	
C5B	0.4588 (7)	0.0224 (4)	0.3197 (3)	0.0289 (12)	
C51B	0.4894 (7)	−0.0828 (4)	0.3850 (3)	0.0289 (12)	
C52B	0.3110 (8)	−0.1094 (5)	0.4517 (3)	0.0383 (17)	
C53B	0.3431 (9)	−0.2075 (5)	0.5136 (3)	0.0433 (17)	
C54B	0.5553 (9)	−0.2817 (4)	0.5088 (3)	0.0368 (14)	
C55B	0.7313 (9)	−0.2594 (5)	0.4423 (3)	0.0420 (17)	
C56B	0.6997 (8)	−0.1610 (5)	0.3807 (3)	0.0386 (17)	
H4A	−0.54410	0.87240	0.10140	0.0300*	
H6A	−0.39030	0.53950	0.23950	0.0330*	
H12A	0.22100	0.52990	0.07550	0.0560*	
H3B	0.18380	0.24180	0.23770	0.0350*	
H21B	0.44930	0.31390	0.11350	0.0410*	
H22B	0.69490	0.24630	0.09820	0.0410*	
H52B	0.16500	−0.05930	0.45460	0.0460*	
H53B	0.22040	−0.22440	0.55940	0.0520*	
H55B	0.87510	−0.31180	0.43860	0.0500*	
H56B	0.82260	−0.14590	0.33460	0.0460*	

### Atomic displacement parameters (Å^2^)

**Table:**

	*U*^11^	*U*^22^	*U*^33^	*U*^12^	*U*^13^	*U*^23^
O2A	0.0266 (15)	0.0346 (17)	0.0350 (17)	0.0035 (14)	0.0012 (13)	0.0106 (13)
O11A	0.0373 (17)	0.0299 (17)	0.0412 (19)	0.0067 (15)	−0.0001 (15)	0.0131 (14)
O12A	0.0308 (17)	0.0316 (17)	0.047 (2)	0.0054 (14)	−0.0018 (15)	0.0162 (14)
O31A	0.0378 (17)	0.0395 (18)	0.0287 (17)	0.0007 (15)	−0.0014 (14)	0.0122 (14)
O32A	0.0308 (16)	0.0224 (16)	0.054 (2)	0.0070 (14)	−0.0025 (15)	0.0143 (14)
O51A	0.0361 (18)	0.0377 (19)	0.050 (2)	0.0159 (16)	0.0058 (15)	0.0097 (16)
O52A	0.0409 (18)	0.043 (2)	0.044 (2)	0.0024 (16)	0.0115 (15)	0.0174 (16)
N3A	0.0237 (19)	0.033 (2)	0.033 (2)	−0.0017 (17)	−0.0063 (17)	0.0097 (17)
N5A	0.030 (2)	0.029 (2)	0.033 (2)	0.0013 (18)	0.0012 (17)	0.0010 (17)
C1A	0.027 (2)	0.019 (2)	0.028 (2)	−0.0007 (18)	−0.0072 (18)	0.0040 (17)
C2A	0.025 (2)	0.025 (2)	0.026 (2)	0.0018 (19)	−0.0040 (18)	0.0008 (18)
C3A	0.027 (2)	0.023 (2)	0.027 (2)	−0.0026 (19)	−0.0055 (18)	0.0093 (17)
C4A	0.024 (2)	0.022 (2)	0.028 (2)	0.0045 (18)	−0.0065 (18)	0.0022 (17)
C5A	0.023 (2)	0.026 (2)	0.025 (2)	0.0037 (18)	−0.0018 (17)	−0.0022 (17)
C6A	0.029 (2)	0.025 (2)	0.028 (2)	−0.0007 (19)	−0.0041 (18)	0.0049 (18)
C11A	0.028 (2)	0.026 (2)	0.034 (3)	0.002 (2)	−0.006 (2)	0.001 (2)
Br1B	0.0977 (5)	0.0413 (3)	0.0352 (3)	−0.0111 (3)	−0.0151 (3)	0.0174 (2)
S1B	0.0254 (6)	0.0321 (6)	0.0341 (6)	0.0043 (5)	0.0000 (5)	0.0122 (5)
N3B	0.0258 (19)	0.0265 (19)	0.032 (2)	0.0067 (16)	−0.0026 (16)	0.0086 (15)
N4B	0.0283 (19)	0.033 (2)	0.028 (2)	0.0002 (17)	0.0002 (16)	0.0068 (16)
N21B	0.0259 (19)	0.034 (2)	0.041 (2)	0.0075 (17)	−0.0015 (17)	0.0154 (17)
C2B	0.026 (2)	0.023 (2)	0.031 (2)	−0.0013 (19)	−0.0047 (19)	0.0041 (18)
C5B	0.031 (2)	0.028 (2)	0.027 (2)	−0.002 (2)	−0.0016 (18)	0.0031 (18)
C51B	0.032 (2)	0.031 (2)	0.024 (2)	−0.007 (2)	−0.0022 (18)	0.0031 (18)
C52B	0.038 (3)	0.039 (3)	0.036 (3)	0.002 (2)	−0.001 (2)	0.000 (2)
C53B	0.048 (3)	0.048 (3)	0.032 (3)	−0.011 (3)	0.005 (2)	0.007 (2)
C54B	0.059 (3)	0.028 (2)	0.025 (2)	−0.009 (2)	−0.009 (2)	0.0066 (19)
C55B	0.047 (3)	0.035 (3)	0.042 (3)	0.006 (2)	−0.006 (2)	0.013 (2)
C56B	0.038 (3)	0.041 (3)	0.033 (3)	0.003 (2)	0.004 (2)	0.015 (2)

### Geometric parameters (Å, º)

**Table:**

Br1B—C54B	1.886 (4)	C1A—C6A	1.387 (6)
S1B—C5B	1.756 (4)	C1A—C11A	1.506 (6)
S1B—C2B	1.720 (4)	C1A—C2A	1.416 (6)
O2A—C2A	1.311 (5)	C2A—C3A	1.409 (6)
O11A—C11A	1.220 (5)	C3A—C4A	1.380 (6)
O12A—C11A	1.296 (5)	C4A—C5A	1.384 (6)
O31A—N3A	1.232 (4)	C5A—C6A	1.374 (6)
O32A—N3A	1.227 (5)	C4A—H4A	0.9500
O51A—N5A	1.222 (5)	C6A—H6A	0.9500
O52A—N5A	1.226 (5)	C5B—C51B	1.461 (6)
O12A—H12A	0.8700	C51B—C56B	1.398 (6)
N3A—C3A	1.456 (6)	C51B—C52B	1.388 (6)
N5A—C5A	1.460 (5)	C52B—C53B	1.376 (7)
N3B—C2B	1.337 (5)	C53B—C54B	1.387 (7)
N3B—N4B	1.360 (4)	C54B—C55B	1.367 (7)
N4B—C5B	1.287 (5)	C55B—C56B	1.375 (7)
N21B—C2B	1.302 (5)	C52B—H52B	0.9500
N3B—H3B	0.8800	C53B—H53B	0.9500
N21B—H22B	0.8800	C55B—H55B	0.9500
N21B—H21B	0.8800	C56B—H56B	0.9500
			
C2B—S1B—C5B	88.1 (2)	O11A—C11A—O12A	124.5 (4)
C11A—O12A—H12A	104.00	O11A—C11A—C1A	121.4 (4)
O32A—N3A—C3A	117.7 (3)	C3A—C4A—H4A	121.00
O31A—N3A—O32A	123.8 (4)	C5A—C4A—H4A	121.00
O31A—N3A—C3A	118.5 (4)	C5A—C6A—H6A	120.00
O51A—N5A—C5A	118.3 (3)	C1A—C6A—H6A	120.00
O51A—N5A—O52A	123.1 (3)	S1B—C2B—N3B	109.7 (3)
O52A—N5A—C5A	118.5 (4)	S1B—C2B—N21B	126.3 (3)
N4B—N3B—C2B	117.4 (3)	N3B—C2B—N21B	124.0 (4)
N3B—N4B—C5B	110.0 (3)	S1B—C5B—N4B	114.8 (3)
C2B—N3B—H3B	121.00	N4B—C5B—C51B	124.5 (4)
N4B—N3B—H3B	121.00	S1B—C5B—C51B	120.7 (3)
C2B—N21B—H21B	120.00	C5B—C51B—C56B	120.4 (4)
H21B—N21B—H22B	120.00	C52B—C51B—C56B	118.2 (4)
C2B—N21B—H22B	120.00	C5B—C51B—C52B	121.3 (4)
C2A—C1A—C11A	120.1 (4)	C51B—C52B—C53B	120.8 (4)
C6A—C1A—C11A	118.9 (4)	C52B—C53B—C54B	119.7 (4)
C2A—C1A—C6A	121.0 (4)	Br1B—C54B—C53B	120.6 (4)
C1A—C2A—C3A	117.0 (4)	C53B—C54B—C55B	120.5 (4)
O2A—C2A—C3A	122.4 (4)	Br1B—C54B—C55B	118.9 (4)
O2A—C2A—C1A	120.6 (4)	C54B—C55B—C56B	119.8 (5)
N3A—C3A—C2A	121.2 (4)	C51B—C56B—C55B	121.0 (4)
N3A—C3A—C4A	116.4 (4)	C51B—C52B—H52B	120.00
C2A—C3A—C4A	122.4 (4)	C53B—C52B—H52B	120.00
C3A—C4A—C5A	118.0 (4)	C52B—C53B—H53B	120.00
N5A—C5A—C4A	117.3 (4)	C54B—C53B—H53B	120.00
N5A—C5A—C6A	120.2 (4)	C54B—C55B—H55B	120.00
C4A—C5A—C6A	122.4 (4)	C56B—C55B—H55B	120.00
C1A—C6A—C5A	119.2 (4)	C51B—C56B—H56B	120.00
O12A—C11A—C1A	114.2 (4)	C55B—C56B—H56B	119.00
			
C2B—S1B—C5B—C51B	179.2 (4)	C11A—C1A—C6A—C5A	178.5 (4)
C2B—S1B—C5B—N4B	−1.5 (3)	O2A—C2A—C3A—N3A	0.1 (6)
C5B—S1B—C2B—N3B	1.5 (3)	O2A—C2A—C3A—C4A	−177.8 (4)
C5B—S1B—C2B—N21B	−178.6 (4)	C1A—C2A—C3A—C4A	1.3 (6)
O32A—N3A—C3A—C2A	−147.8 (4)	C1A—C2A—C3A—N3A	179.2 (4)
O31A—N3A—C3A—C4A	−148.7 (4)	C2A—C3A—C4A—C5A	−1.1 (6)
O32A—N3A—C3A—C4A	30.3 (5)	N3A—C3A—C4A—C5A	−179.0 (4)
O31A—N3A—C3A—C2A	33.3 (6)	C3A—C4A—C5A—N5A	−179.1 (4)
O51A—N5A—C5A—C4A	−1.1 (6)	C3A—C4A—C5A—C6A	0.3 (6)
O51A—N5A—C5A—C6A	179.5 (4)	C4A—C5A—C6A—C1A	0.1 (7)
O52A—N5A—C5A—C4A	178.0 (4)	N5A—C5A—C6A—C1A	179.5 (4)
O52A—N5A—C5A—C6A	−1.5 (6)	S1B—C5B—C51B—C52B	178.1 (4)
N4B—N3B—C2B—N21B	178.7 (4)	S1B—C5B—C51B—C56B	−3.0 (6)
C2B—N3B—N4B—C5B	0.3 (5)	N4B—C5B—C51B—C52B	−1.1 (7)
N4B—N3B—C2B—S1B	−1.4 (4)	N4B—C5B—C51B—C56B	177.8 (4)
N3B—N4B—C5B—C51B	−179.8 (4)	C5B—C51B—C52B—C53B	−179.1 (4)
N3B—N4B—C5B—S1B	0.9 (4)	C56B—C51B—C52B—C53B	2.0 (7)
C6A—C1A—C2A—C3A	−0.8 (6)	C5B—C51B—C56B—C55B	179.3 (4)
C6A—C1A—C2A—O2A	178.3 (4)	C52B—C51B—C56B—C55B	−1.7 (7)
C2A—C1A—C6A—C5A	0.2 (6)	C51B—C52B—C53B—C54B	−0.7 (7)
C11A—C1A—C2A—O2A	0.0 (6)	C52B—C53B—C54B—Br1B	178.7 (4)
C11A—C1A—C2A—C3A	−179.1 (4)	C52B—C53B—C54B—C55B	−1.0 (7)
C2A—C1A—C11A—O11A	−178.0 (4)	Br1B—C54B—C55B—C56B	−178.4 (4)
C2A—C1A—C11A—O12A	2.2 (6)	C53B—C54B—C55B—C56B	1.3 (7)
C6A—C1A—C11A—O11A	3.7 (6)	C54B—C55B—C56B—C51B	0.1 (7)
C6A—C1A—C11A—O12A	−176.2 (4)		

### Hydrogen-bond geometry (Å, º)

**Table:**

*D*—H···*A*	*D*—H	H···*A*	*D*···*A*	*D*—H···*A*
O12*A*—H12*A*···O2*A*	0.87	1.57	2.418 (4)	164
N3*B*—H3*B*···O11*A*	0.88	1.87	2.744 (4)	172
N21*B*—H21*B*···O12*A*	0.88	1.89	2.747 (4)	166
N21*B*—H22*B*···O2*A*^i^	0.88	2.22	2.897 (4)	134
N21*B*—H22*B*···O31*A*^i^	0.88	2.19	2.986 (5)	150
C4*A*—H4*A*···O32*A*^ii^	0.95	2.44	3.284 (5)	148
C56*B*—H56*B*···O51*A*^iii^	0.95	2.44	3.364 (5)	164
C56*B*—H56*B*···S1*B*	0.95	2.64	3.081 (5)	109

Symmetry codes: (i) −*x*+1, −*y*+1, −*z*; (ii) −*x*−1, −*y*+2, −*z*; (iii) *x*+2, *y*−1, *z*.
